# The relationship of coping skills with psychache in patients with depressive disorder

**DOI:** 10.1097/MD.0000000000034339

**Published:** 2023-07-21

**Authors:** Caner Yeşiloğlu, Lut Tamam, Mehmet Emin Demirkol, Zeynep Namli, Mahmut Onur Karaytuğ, Şilan Şenbayram Güzelbaba

**Affiliations:** a Kirşehir Training and Research Hospital, Department of Psychiatry, Kirşehir, Turkey; b Çukurova University School of Medicine, Department of Psychiatry, Adana, Turkey; c Ömer Halisdemir University Training and Research Hospital, Niğde, Turkey.

**Keywords:** coping, demoralization, mental pain, suicidal behavior, suicide, tolerance for psychache

## Abstract

Suicide is a leading cause of death and disability worldwide. Psychache (psychological pain) and diminished tolerance of psychaches are important risk factors for suicide. People experiencing psychaches of similar severity may not demonstrate the same levels of tolerance because of various coping skills. This study aimed to determine the relationship between psychache, tolerance for psychache, and coping skills in individuals with depression and healthy controls. We included 73 patients with depressive disorders without comorbid mental disorders and 65 healthy controls. We applied beck depression inventory, beck hopelessness scale, beck suicidal ideation scale, psychache scale, tolerance for mental pain scale (TMPS), and coping attitudes evaluation scale (COPE) to all participants. People with depression had significantly higher COPE dysfunction scores than those in the control group did. Patients who had previously attempted suicide attempt(s) previously had significantly higher beck hopelessness scale, beck depression inventory, COPE dysfunction, and psychache scale scores, and lower TMPS-10 scores than those who did not attempt suicide. Mediation analyses revealed that dysfunctional coping skills played a partial mediating role in the relationship between psychache and the TMPS. The study revealed that dysfunctional coping skills were related to suicidal ideation and previous suicide attempts. These findings suggest that improving coping skills could help reduce the severity of suicidal ideation.

## 1. Introduction

According to the World Health Organization, approximately 14 million people attempt suicide every year, of which more than 700,000 die.^[1,2]^ Predicting suicidal behavior remains a challenge, despite certain known risk factors such as psychiatric disorders and previous suicide attempts (SA);^[3]^ socioeconomic factors such as unemployment, juvenescence, and sex (being female); and psychological factors such as psychache (psychological pain, mental pain) and hopelessness.^[4]^

Shneidman^[5]^ characterized psychache as a real or idealized loss, leading to a painful state with repercussions for self-esteem and integrity. After reviewing suicide notes, Shneidman^[5]^ suggested that suicide is considered a remedy for escaping intensive psychaches, and that there would be no suicide without psychache. Although psychache is generally related to mental disorders, individuals without mental disorders may experience it.^[6]^ Risk factors, such as depression, hopelessness, childhood trauma, and sleep disturbances, increase the risk of suicide through psychache.^[7,8]^ Meerwijk et al^[9]^ found that the inability to tolerate psychache is associated with suicidality more than its intensity, and that tolerance for psychache depends on the use of various coping skills. Previous studies have suggested evaluating coping skills to prevent suicide and determining appropriate treatment approaches for patients with depression experiencing psychache.^[9–11]^

Coping strategies refer to an individual’s behavioral and cognitive patterns when facing external and internal challenges. Coping strategies initiate, maintain, and modulate affective responses to stressful events. Coping strategies can be categorized as adaptive, maladaptive, problem-focused, emotion-focused, or dysfunctional. The presence of dysfunctional emotion regulation strategies is more significant than the absence of functional coping mechanisms in the etiology of mental disorders.^[12]^ Attitudes toward solving the source of the difficulty involve problem-focused coping skills; for solving or sharing individual feelings, it is emotion-focused coping skills; and those that are not inclined to adaptation are dysfunctional coping skills.^[13,14]^ While dysfunctional coping skills negatively affect prognosis by increasing the severity of psychiatric complaints, problem-focused strategies can reduce symptom severity.^[15,16]^ To the best of our knowledge, no previous study has investigated the role of coping skills in the development of tolerance to psychache. We aimed to investigate the relationship between psychache, tolerance for psychache, and coping skills in patients with major depressive disorder (MDD) and healthy controls. We hypothesized that coping skills would mediate the relationship between psychache and tolerance to psychache. Our secondary hypothesis was that patients with MDD would use dysfunctional coping skills more than healthy controls.

## 2. Subjects and methods

### 2.1. Sample

**Figure 1.**
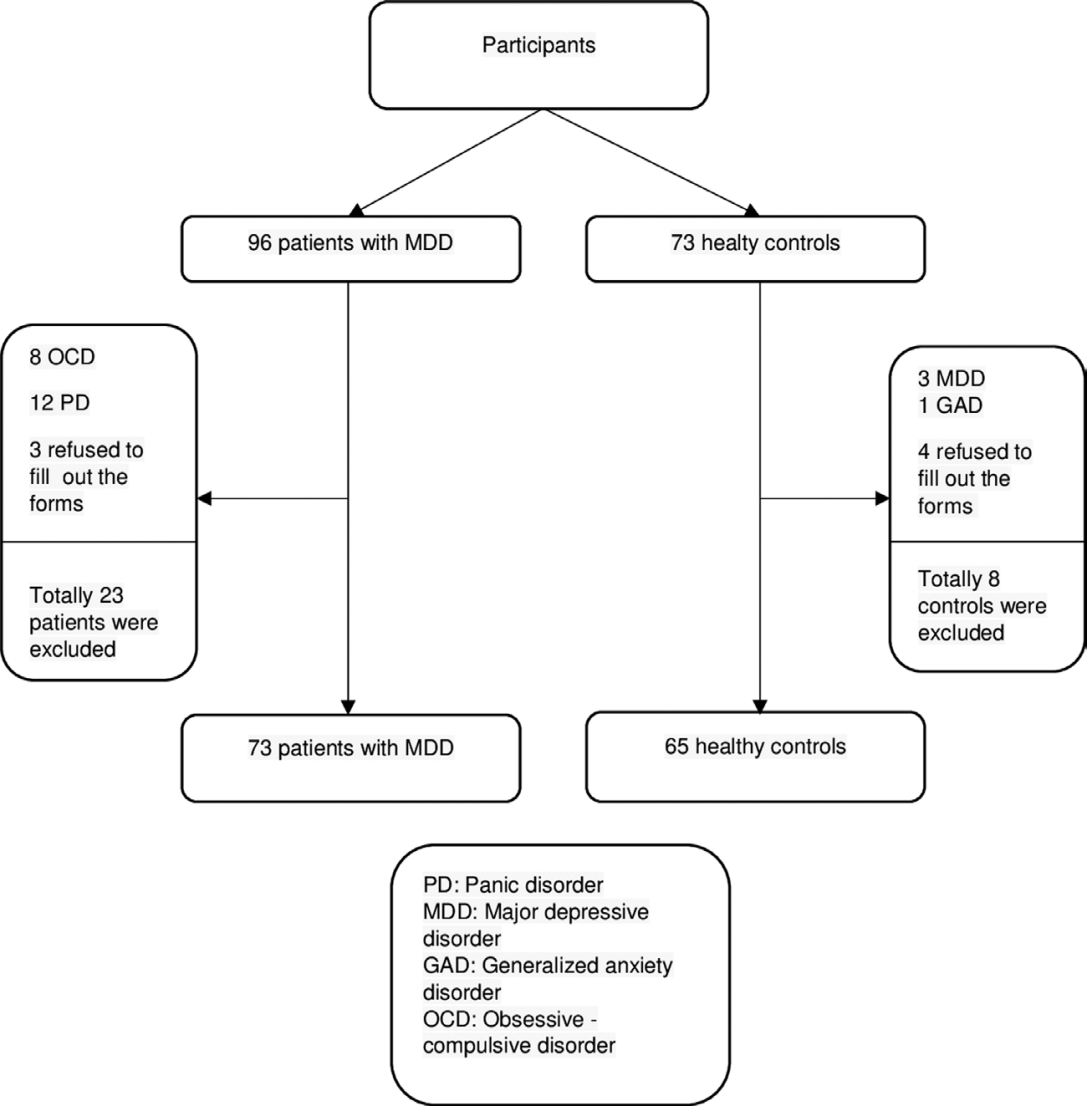
Number of participants and exclusion criteria.

We included 96 literate patients with MDD, according to the fifth edition of the diagnostic and statistical manual of mental disorders criteria, aged between 18 and 65 years who were admitted to Cukurova University Faculty of Medicine Balcali Hospital, and 73 healthy controls who had not been diagnosed with psychiatric disorders earlier, had not used any psychotropic drugs, and had sociodemographic characteristics similar to those of the depression group (Figure 1). Cukurova University Faculty of Medicine Balcali Hospital is a tertiary hospital located in southern Turkey that serves a population of 3 million per year. The first author conducted psychiatric interviews with all participants for 40 to 60 minutes using the Structured Clinical Interview for fifth edition of the diagnostic and statistical manual of mental disorders disorders-clinician version. For better comprehension, the interviewer explained the items to participants. The scales were applied to interview participants for which the diagnoses were determined. To complete the self-report questionnaires, participants were required to be literate. The clinician completed the sociodemographic data form prepared during the interviews. In this study, the coping attitudes evaluation scale (COPE), psychache scale (PS), tolerance of mental pain scale (TMPS), beck depression inventory (BDI), beck hopelessness scale (BHS), and beck suicidal ideation scale (BSIS) in the native language (Turkish) were completed under the supervision of a clinician. Eight participants in the patient group diagnosed with obsessive-compulsive disorder and 12 with panic disorder were excluded from the study to eliminate the confounding effects of comorbid conditions. Three patients were excluded as they did not complete the questionnaire. In the control group, 3 participants diagnosed with MDD, 1 with generalized anxiety disorder, and 4 who failed to complete the scale were excluded from the study. This study included 73 patients with MDD and 65 healthy volunteers. The Cukurova University Faculty of Medicine nonInvasive Clinical Research Ethics Committee approved this study (Decision No. January 95 10, 2020). All the participants provided written informed consent. This study was conducted in accordance with the principles of the Declaration of Helsinki.^[17]^

The sample size (power analysis) of the study was calculated using the G Power 3.1 program. With a medium effect size (Cojen d = 0.50), power of 0.80 and margin of error of 0.05 (*P* = .05), the minimum sample size required in a single group was calculated as 64 for the patient and control groups.

### 2.2. The sociodemographic data form

Participants’ age, gender, educational level, employment status, marital status, place of residence, and history of SA were queried using this form.

### 2.3. Coping attitudes evaluation scale

The COPE is a Likert-type self-report scale that consists of 60 questions. The answers ranged from “I usually do not do this at all” to “I usually do this a lot” (1–4). Carver et al^[14]^ defined the 3 coping skills in COPE as problem-focused, emotion-focused, and dysfunctional. Active coping efforts, social support, and planning were defined as problem-focused coping skills. Acceptance, emotional support, humor, positive reinterpretation, and holding on to religious beliefs are considered emotion-focused coping skills, and behavioral disconnection, denial, distraction, self-blame, and asubstance use are dysfunctional coping skills.^[14]^ In the Turkish version’s validity and reliability study, Cronbach alpha coefficient was 0.79,^[18]^ while, and 0.74, respectively.

### 2.4. Psychache scale

The PS is a self-report scale consisting of 13 questions that evaluate psychache as the process of mental suffering that occurs due to inadequacy or lack of self, or not satisfying needs such as love, attachment, success, and avoiding harm. The answers were within the range of “Never/Strongly disagree” to “Always/strongly agree”. Higher scores indicate more severe and frequent psychaches.^[19]^ Demirkol et al^[20]^ revealed that the Turkish version’s Cronbach alpha value was 0.98, while it was 0.96 in our study.

### 2.5. Tolerance of mental pain scale

The TMPS-10 is a 5-point Likert-type scale that evaluates tolerance to psychaches. Responses ranged from 1 (not true) to 5 (very true).^[9]^ Increased scale scores indicated that individuals tolerated the psychache better. In our study, the Cronbach alpha value of the Turkish version was 0.98,^[10]^ whereas it was 0.78 in our study. The original version of the Meerwijk scale includes 2 subdimensions: managing and enduring mental pain. However, the Turkish version revealed that the 2-factor structure could be considered a single factor because of the strong correlation between them.^[10]^

### 2.6. Beck depression inventory

The BDI is a 4-point Likert-type self-report scale consisting of 21 items that measure the severity of depressive symptoms. Higher scores indicate more severe depressive symptoms.^[21]^ Hisli et al^[22]^ found a Cronbach alpha value of 0.80 in a Turkish validity and reliability study, while it was 0.79 in our study.

### 2.7. Beck hopelessness scale

The BHS is a self-reported scale consisting of 20 items that determine hopelessness levels. The questions were answered with “yes” or “no.” Higher total scores indicate increased hopelessness.^[23]^ Seber et al^[24]^ revealed that the Cronbach alpha coefficient was 0.86 in a Turkish adaptation study, while it was 0.84 in our study.

### 2.8. Beck suicidal ideation scale

BSIS was developed to assess the dimensionality of suicidal ideation (SI) and its clinical implications. It consists of 5 components: evaluating attitudes toward life or death, features of suicidal intent, characteristics of the planned attempt, realization of the planned attempt, and background factors. Each item on this scale was scored between 0 and 2. Higher total scores indicate more severe SI.^[25]^ The Cronbach alpha value was 0.84 in the Turkish validity and reliability study of the form;^[26]^ it was 0.82 in our study.

### 2.9. Statistical analysis

Descriptive statistics were summarized as percentages and numbers for categorical variables. The groups were considered to be normally distributed when the skewness and kurtosis values were between −1.5 and + 1.5.^[27]^ When the continuous variables were distributed normally, they were presented as mean ± standard deviation, and when not, as median and quartiles. For comparison of categorical variables, the chi-squared test was used. To compare the means of 2 independent groups, the independent samples t-test was used when the data showed normal distribution, and the Mann–Whitney *U* test when not. Pearson Rho correlation coefficient (2-tailed) was used when the distribution of continuous variables was normal. Multivariate logistic regression analysis was used to evaluate the risk factors related to a history of SA. The total TMPS score was excluded from regression analysis because of multicollinearity.

Mediation analyses were performed using the bootstrap method (bootstrap 5000), the maximum likelihood parameter estimation method, and continuous variables. First, we tested whether the external (exogenous) variable (psychache) significantly predicted the internal (endogenous) variable (tolerance for psychache) in the basic model without including a mediator variable in the analysis. We investigated direct and indirect effects after including the mediator. Jamovi (Version 1.8.1) (Jamovi project 2018, retrieved from https://www.jamovi.org) and MPLUS 7.4 programs were used for statistical analysis. The significance level was set at *P* = .05 (*P* value).

## 3. Results

The depression and control groups were similar in terms of age, sex, place of residence, and educational, occupational, and marital status. Table 1 presents the participants sociodemographic characteristics.

**Table 1:** Sociodemographic and clinical characteristics of the participants.

	Depression n (%)	Control n (%)	*P* value
Gender			.42*
Female	45 (61.6)	36 (55.3)	
Male	28 (38.4)	29 (44.7)	
Marital status			.23*
Single	29 (39.7)	32 (49.2)	
Married	44 (60.3)	33 (50.8)	
Occupation			.14*
Unemployed	32 (43.8)	35 (53.9)	
Employed	41 (56.2)	30 (46.1)	
Place of residence			.26†
Urban	45 (61.6)	45 (69.3)	
Rural	28 (38.4)	20 (30.7)	
Previous suicide attempts			**.001** *
Absent	56 (76.7)	65 (100)	
Present	17 (23.3)	0 (0)	
Age	39.85 ± 11.76	38.40 ± 10.19	.45†
Years of education	10.77 ± 3.59	11.53 ± 3.83	.23†

The *p* values in bold are statistically significant (*P* < .05).

* Chi-square.

† Independent samples *t* test.

In the depression group, BDI, BSIS, BHS, PS were significantly higher, whereas TMPS-10 scores were significantly lower than those in the control group (*P* < .05 each). In the depression group, COPE dysfunctional scores were significantly higher, whereas COPE problem-focused scores were significantly lower than those in the control group (*P* < .05 each). There is no significant difference between the groups in emotion-focused coping scores. Table 2 presents the BDI, BSIS, BHS, TMPS-10, PS, and COPE scores.

**Table 2:** Comparison of the groups scale scores.

Variables	Patient	Control	*P* value
BHS	7 (3–13.5)	1 (0–2)	**<.001** *
BDI	30 (15–36.5)	2 (0–5)	**<.001** *
BSIS	3 (0–7)	0 (0–0)	**<.001** *
PS	40.71 ± 11.53	20.95 ± 7.79	**<.001** †
TMPS-10	33.96 ± 5.21	35.76 ± 5.49	**<.05** †
COPE dysfunctional	48 (42–52.5)	42 (37–44)	**<.001** *
COPE emotion-focused	52.37 ± 9.53	51.55 ± 10.87	.64†
COPE problem-focused	31.27 ± 6.75	35.11 ± 6.86	**<.001** †

The *p* values in bold are statistically significant (*P* < .05).

BHS = beck hopelessness scale, BDI = beck depression inventory, BSIS = beck suicidal ideation scale, COPE = cope inventory, PS = psychache scale, TMPS-10 = tolerance of mental pain scale.

* Mann–Whitney *U*.

† Independent groups *t* test.

Table 3 presents the correlations between the COPE dysfunctional subscale, COPE problem-focused subscale, COPE emotion-focused subscale, and the other scales in the depression group. There was a significant positive correlation between the COPE dysfunctional subscale and the BHS (*P* = .049, *R* = 0.231), BDI (*P* = .022, *R* = 0.267), BSIS (*P* = .008, *R* = 0.308), and PS scores (*P* = .010, *R* = 0.299) and a significant negative correlation between the TMPS-10 scores (*P* = .000, r = −0.498).

**Table 3:** Correlations between the scale scores in the depression group.

		COPE emotion-focused	COPE dysfunctional	COPE problem-focused
BHS	Pearson Correlation	−.220	.231	−.147
	Sig.	**.062**	**.049**	.213
BDI	Pearson Correlation	−.374	.267	−.364
	Sig.	**.001**	**.022**	**.002**
BSIS	Pearson Correlation	−.120	.308	−.193
	Sig.	.312	**.008**	.101
PS	Pearson Correlation	−.391	.299	−.302
	Sig.	**.001**	**.010**	**.009**
TMPS−10	Pearson Correlation	.221	−.498	.253
	Sig.	.061	**.000**	**.031**

The values in bold are statistically significant (*P* < .05).

BHS = beck hopelessness scale, BDI = beck depression inventory, BSIS = beck suicidal ideation scale, PS = psychache scale, Sig = significant, TMPS-10 = tolerance of mental pain scale.

There was a negative correlation between problem-focused COPE and BDI (*P* = .002, r = −0.364) and PS scores (*P* = .009, r = −0.302) and a positive and significant correlation between TMPS-10 scores (*P* = .031, *R* = 0.253). There was also a negative correlation between COPE emotion focus and the BDI (*P* = .001, r = −0.374) and PS scores (*P* = .001, r = −0.391).

Table 4 compares the scale scores of patients with depression, with and without a history of SA. The median BHS, BDI, BSIS, and COPE dysfunctional scores were significantly higher in participants with a history of SA than in those without (*P* < .001, *P* < .001, *P* < .001, and *P* = .004, respectively). The median PS score of those with a history of SA was significantly higher, while the median TMPS-10 scores were lower than those of those without (*P* = .02, *P* = .002, respectively). The groups had similar COPE emotion and problem-focused scores (*P* = .3, *P* = .85, respectively).

**Table 4:** The relationship between previous suicide attempt(s) and scale scores.

	Previous suicide attempts		
	Absent	Present	
BHS	7.20 ± 5.64	12.35 ± 4.66	***P* < .001** *
BDI	23.82 ± 12.99	36.94 ± 9.36	***P* < .001** *
BSIS	2.57 ± 2.90	9.11 ± 2.99	***P* < .001** *
PS	38.95 ± 11.61	46.53 ± 9.42	***P* = .02** *
TMPS-10	34.96 ± 4.71	30.65 ± 5.57	***P* = .002** *
COPE disfunctional	45.79 ± 6.60	57.35 ± 14.01	***P* = .004** *
COPE emotion-focused	53.01 ± 9.05	50.24 ± 10.98	*P* = .3*
COPE Problem-Focused	31.36 ± 6.84	31 ± 6.63	*P* = .85*

The *p* values in bold are statistically significant (*P* < .05).

BHS = beck hopelessness scale, BDI = beck depression inventory, BSIS = beck suicidal ideation scale, PS = psychache scale, TMPS-10 = tolerance for mental pain scale, COPE = cope inventory.

* Independent samples *t* test.

There was a weak positive correlation between the COPE emotion and problem-focused subscales (*P* = .001, *R* = 0.383). There was no significant correlation between the dysfunctional COPE and problem-focused (*P* = .380, *R* = 0.104) or emotion-focused (*P* = .247, *R* = 0.137) subscales.

When the scale scores and sociodemographic characteristics were compared, the COPE dysfunctional scores were higher in employed participants than in unemployed participants (*P* < .05). The mean COPE problem-focused scores were significantly higher in male participants than in female participants and higher in employed than unemployed participants (*P* < .05).

In the depression group, there was a negative correlation between age and mean BSIS scores (*P* = .02), and a positive correlation between COPE problem-focused scores (*P* = .02). However, there was no correlation between the PS, TMPS, COPE dysfunction, and COPE emotion-focused scores.

There was a negative correlation between the mean years of education and PS (*P* = .01) and a positive correlation between the COPE dysfunction (*P* = .02) scores. However, there was no correlation between the BSIS, TMPS, COPE emotion-focused, and COPE problem-focused scores.

There was a positive correlation between years of depression and the mean PS score (*P* < .001) and a negative and significant correlation between COPE problem-focused scores (*P* = .02), TMPS (*P* = .04), and COPE dysfunctional score (*P* = .04). There was no correlation between years of depression and the mean BSIS and COPE emotion-focused scores.

Table 5 presents the mediation analysis results for the mediating role of dysfunctional coping skills in the relationship between psychache and tolerance for psychache in patients with depression. The basic model (Model I) revealed that PS scores had a significant and negative relationship with TMPS-10 scores (β = −0.13; *P* = .01), excluding the mediator variable (dysfunctional coping skills). There was a negative and significant relationship between psychache and tolerance for psychache in Model II, in which dysfunctional coping skills were included (β = −0.13; *P* = .01). In summary, psychache was significantly related to psychache tolerance in patients with depression in the basic model (*P* = .01). In the mediation analysis, in which dysfunctional coping skills (mediator) were included, the indirect (*P* = .02) and direct relationships (*P* = .01) were significant. Therefore, it was concluded that dysfunctional coping skills played a partial mediating role in the relationship between psychache and tolerance in patients with depression (Table 5).

**Table 5:** The mediating effect of dysfunctional coping skills in the relationship between psychache and the tolerance for psychache.

Model	Path	Coefficient	Standard Error	*P* value
I. Simple model (without M)	X →Y	−0.13	0.05	**0.01**
II. Mediation analysis	X → M	0.26	0.1	**0.01**
II. Mediation analysis	M→Y	−0.21	0.05	**0.0002**
II. Mediation analysis (direct effect)	X →Y	−0.13	0.05	**0.01**
II. Mediation analysis (indirect effect)	Path	Coefficient	Standard error	Bootstrap Confidence interval BootLower limit/BootUpper limit
	X→ M→Y	−0.06	0.02	−0.104/−**0.02**

The *p* values in bold are statistically significant (*P* < .05).

X = psychache scale, M = dysfunctional coping skills, Y = tolerance for mental pain scale-10.

**Figure 2.**
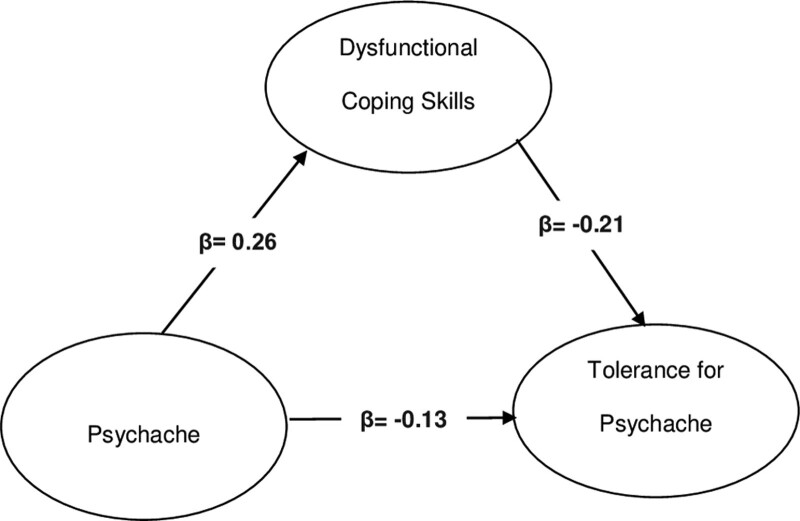
The path diagram for mediation analysis between psychache, tolerance for psychache, and dysfunctional coping skills.

Figure 2 presents the path diagram, which includes the standardized path coefficients. There was a positive and significant relationship between psychache and dysfunctional coping skills (β = 0.26; *P* = .01), and a negative and significant relationship between dysfunctional coping skills and tolerance for psychache (β = −0.21; *P* = .0002). Accordingly, it was concluded that the direct and indirect relationships in the path of psychache, dysfunctional coping skills, and tolerance for psychache, respectively, were negative and significant (β = −0.06; *P* = .02).

Table 6 presents the factors that influenced the history of SA. According to the logistic regression analysis, these factors were sex, BDI, and COPE dysfunction scores. The model complied with the data according to the Hosmer–Lemeshow test (*P* > .05). The classification accuracy was 86.3%. Independent variables accounted for 59% of the suicides. According to the model, females were 0.12 times less likely to commit suicide than males, a 1-unit rise in BDI scores increases the probability of suicide 1.11 times, and a 1-unit increase in COPE dysfunctional scores raises the probability of suicide 1.16 times.

**Table 6:** Comparison of the effective factors on suicide.

	B	SE	Wald	Df	*P* value	Exp(B)
Gender	−2.138	.983	4.731	1	**.030**	.118
BDI	.102	.050	4.186	1	**.041**	1.107
COPE problem-focused	−.072	.079	.831	1	.362	.931
PS	−.030	.051	.346	1	.557	.970
COPE dysfunctional	.149	.054	7.453	1	**.006**	1.160
BHS	.112	.077	2.125	1	.145	1.118
Marital status	.962	.852	1.274	1	.259	2.616
Constant	−8.713	3.846	5.133	1	**.023**	.000

The *p* values in bold are statistically significant (*P* < .05).

Sig = significancy, B = coefficient, COPE = coping attitudes evaluation scale, SE = standard error, Wald = wald statistics value, Df = degree of freedom, BHS = beck hopelessness scale, BDI = beck depression inventory, PS = psychache scale.

## 4. Discussion

Suicide is the most important preventable cause of death worldwide.^[28]^ The risk of suicide increases with unbearable psychaches resulting from negative emotions, such as hopelessness, demoralization, shame, fear, guilt, loneliness, and frustration.^[29,30]^ Coping skills play an essential role in reducing the negative effects of psychache, with the threshold of psychache varying among individuals.^[5,29]^ The most important outcome of this study was that the risk of suicide increased in patients with depression who experienced unbearable psychache and used dysfunctional coping skills.

Demoralization is the feeling of helplessness and hopelessness due to inadequate coping skills and increases the risk of suicide independent of mental disorders.^[30,31]^ Meerwijk et al^[9]^ found that individuals who attempted suicide used problem-focused coping skills to suppress the source of the problem and emotion-focused coping skills for emotions caused by stressful situations less frequently than did healthy individuals. Meerwijk et al^[9]^ also found that individuals with a previous SA had a lower tolerance to psychache than those without an SA. Our study found that individuals with a history of SA and those who did not use problem- or emotion-focused coping skills had similar rates. However, our study also revealed that individuals who attempted suicide used dysfunctional coping skills more often than those who did not, and those with a history of SA had lower tolerance for psychache than those without SA. These results suggest that dysfunctional coping strategies do not reduce individuals’ stress levels, contribute to solutions or adaptations to mental problems, or develop tolerance for psychaches.

Li et al^[29]^ demonstrated a strong correlation between SI, psychache, and depression in university students. Troister and Holden^[32]^ found that university students SI and attempts increased with the intensity of their psychache, depression, and hopelessness. Patients with depression often experience inadequacy or worthlessness. Individuals move away from a solution and adopt dysfunctional methods when they predict that they cannot eliminate the source of stress.^[33,34]^ Our study revealed that dysfunctional coping skills increased with severity of depressive symptoms. Our regression model supports the findings of previous studies by demonstrating that SA are closely related to dysfunctional coping skills and depressive symptoms. Dysfunctional coping skills, such as self-blame and substance use, are known risk factors for suicide;^[14,35]^ therefore, our finding that individuals who use dysfunctional coping skills are more likely to attempt suicide is not surprising.

Becker et al^[36]^ found that depressive symptoms, hopelessness, and SI increase as psychache tolerance decreases. Suzuki et al^[16]^ reported that psychiatric complaints were more common in 2259 randomly selected Japanese participants who used emotion-focused and dysfunctional coping skills more intensively than in those who did not. We found that dysfunctional coping skills, psychache severity, and hopelessness intensity correlated in the same manner. Although coping skills are classified as emotion-focused, problem-focused, and dysfunctional, these strategies are not independent and are generally used together in stressful situations. Clinical representation is determined in line with the coping strategies that are used more intensively.^[13,14,33]^ Regier and Parmele^[37]^ showed that those who used dysfunctional coping strategies displayed increasing symptoms of depression. Moreover, Regier and Parmele^[37]^ revealed that the severity of disability and depression increases in individuals with chronic diseases, and that they use emotion-focused skills more frequently than problem-focused skills. The development of strategies to address this problem is one of the most effective coping strategies.^[38]^ Not using problem-focused coping skills may complicate problems and exacerbate depressive symptoms.^[39]^ Our study concluded that individuals who used dysfunctional coping skills displayed more severe depressive symptoms than did those who did not. Impairment in adaptation to stressful situations through dysfunctional coping skills may lead to a negative evaluation of the future and an increase in depressive symptoms.^[16]^

McGarry et al^[15]^ stated that problem-focused strategies and emotion-focused coping skills are used more intensively, and dysfunctional coping skills are used less as occupational experience increases. Our study revealed that employed participants used problem-focused coping skills more than unemployed participants did, which was considered a result of being obliged to find solutions to problems in the workplace. McGarry et al^[15]^ found that resilience to stressful events increased among health care professionals caring for older children. McGarry et al^[15]^ associated these findings with life experiences and improvement in coping skills. Similarly, Meerwijk et al^[9]^ revealed that psychache was better tolerated by older age groups, and interpreted their findings as increased self-confidence in life experiences. In contrast, Demirkol et al^[28]^ found a significant correlation between age and tolerance for psychache in healthy individuals but not in patients with depression. Our results showed that tolerance for psychache did not advance as the number of years of depressive disorder increased, which is similar to the findings of Demirkol et al^[28]^ findings. These results can be attributed to the tolerance of mental pain and an increase in psychological resilience with age in healthy individuals, similar to the findings of Gooding et al^[40]^ findings. The cultural diversity in coping mechanisms may explain the different results of studies conducted in diverse societies.^[41]^

This study had several clinical outcomes. We found that psychache, tolerance for psychache, and dysfunctional coping skills were associated with SA in patients with depression and that dysfunctional coping skills mediated the relationship between psychache and tolerance of psychache. CBT has been proven to be effective in the treatment of psychiatric disorders and also in suicidal tendencies as monotherapy or combination with drugs.^[42]^ Hamdan-Mansour et al^[43]^ showed that individuals with depressive disorders began to use dysfunctional coping skills less frequently and the severity of their depressive symptoms decreased after cognitive behavior therapy sessions. Our results suggest that therapeutic interventions for improving coping skills may help individuals at a high-risk of suicide.

Some of the limitations of this study are that it had a cross-sectional design and was conducted in a university hospital. Furthermore, we did not categorize patients with depression as having a history of psychotherapy or as those who did not. We also did not evaluate the effects of coping skills on the treatment responses. Evaluating the relationship between coping skills and suicidality in patients with depression but not in the healthy population can be considered a strong point of our study. Additionally, it determined not only the direct relationship between coping skills and psychache and tolerance for psychache but also indirect effects. The exclusion of patients with anxiety and obsessive-compulsive disorders owing to confounding effects could be considered another strength of this study. Prospective longitudinal studies assessing the relationship between coping skills and other psychiatric disorders will help to determine the role of coping skills in the emergence and clinical course of psychopathology. Studies with larger samples, including culturally different individuals, are required to better understand this issue.

## 5. Conclusion

Predicting suicide continues to be a challenge despite certain sociodemographic and psychological risk factors. Determining the specific risk factors for suicide may help identify high-risk individuals. Our study showed that tolerance for psychache, a protective factor against suicide, is negatively affected by dysfunctional coping skills. These findings suggest that improving coping skills could help reduce the severity of SI.

## Author contributions

**Conceptualization:** Caner Yesiloglu, Lut Tamam, Mehmet Emin Demirkol.

**Data curation:** Caner Yesiloglu, Mehmet Emin Demirkol, Mahmut Onur Karaytuğ.

**Formal analysis:** Caner Yesiloglu, Mehmet Emin Demirkol, Mahmut Onur Karaytuğ.

**Investigation:** Caner Yesiloglu, Mehmet Emin Demirkol.

**Methodology:** Caner Yesiloglu, Zeynep Namli, Mahmut Onur Karaytuğ.

**Resources:** Caner Yesiloglu, Mahmut Onur Karaytuğ.

**Software:** Caner Yesiloglu, Zeynep Namli, Mahmut Onur Karaytuğ.

**Supervision:** Lut Tamam, Mehmet Emin Demirkol.

**Writing – original draft:** Caner Yesiloglu, Şilan Şenbayram Güzelbaba.

**Writing – review & editing:** Caner Yesiloglu, Lut Tamam, Mehmet Emin Demirkol, Şilan Şenbayram Güzelbaba.
